# Supplementary material for: Evaluating the utility of identity-by-descent segment numbers for relatedness inference via information theory and classification
Source: G3 (Bethesda). 2022 Mar 28;12(6):jkac072. doi: 10.1093/g3journal/jkac072 (PMC9157175; doi:10.1093/g3journal/jkac072)
Supplement: jkac072_Supplementary_Data [file jkac072_supplementary_data.pdf]

Supplement for Evaluating the utility of identity-by-descent segment numbers for relatedness inference via information theory and classification

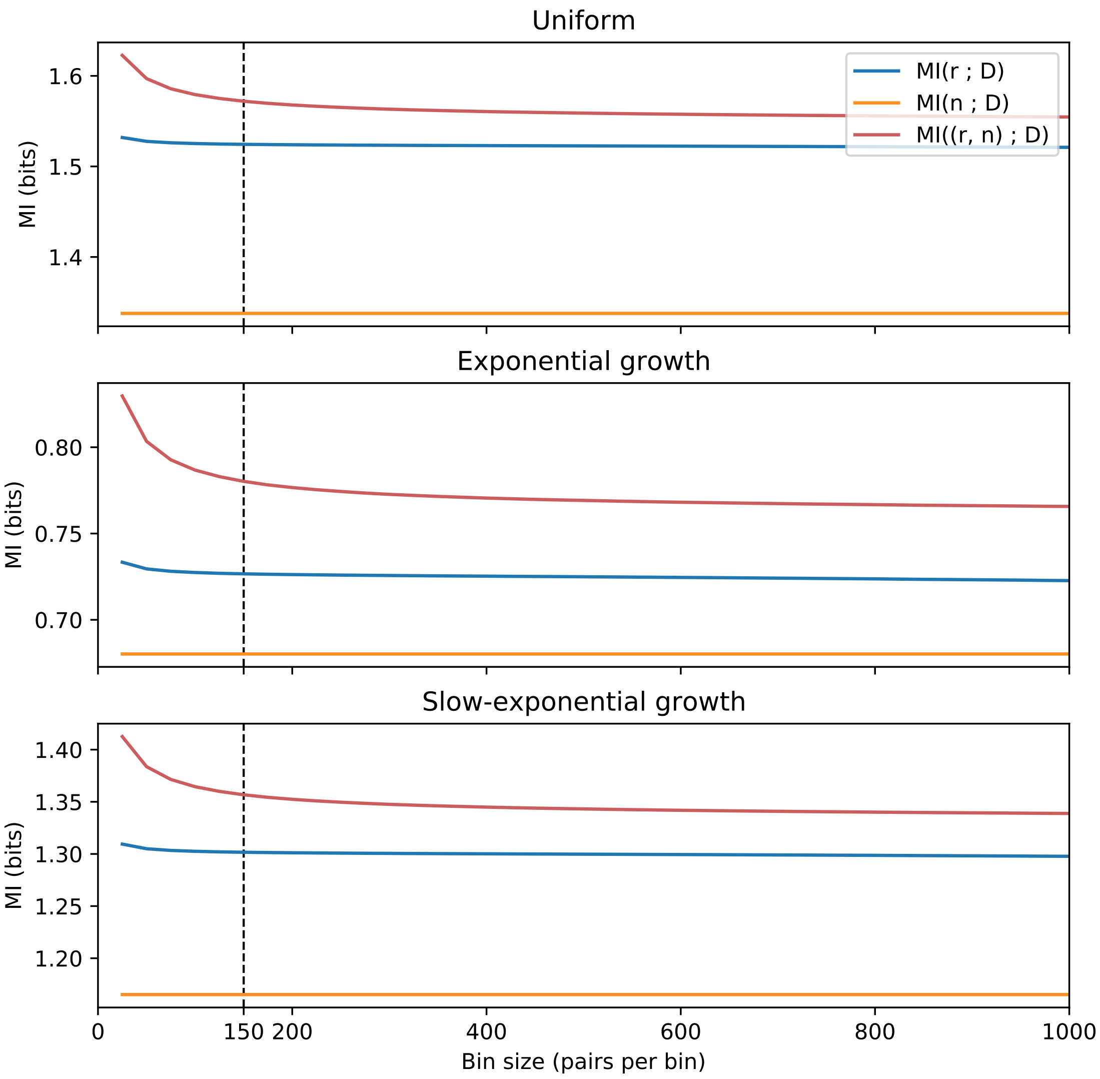

Figure S1: MI of different feature sets as a function of bin size (pairs per bin), averaged over 80 independent simulations of exact segments from each of the distribution shapes.

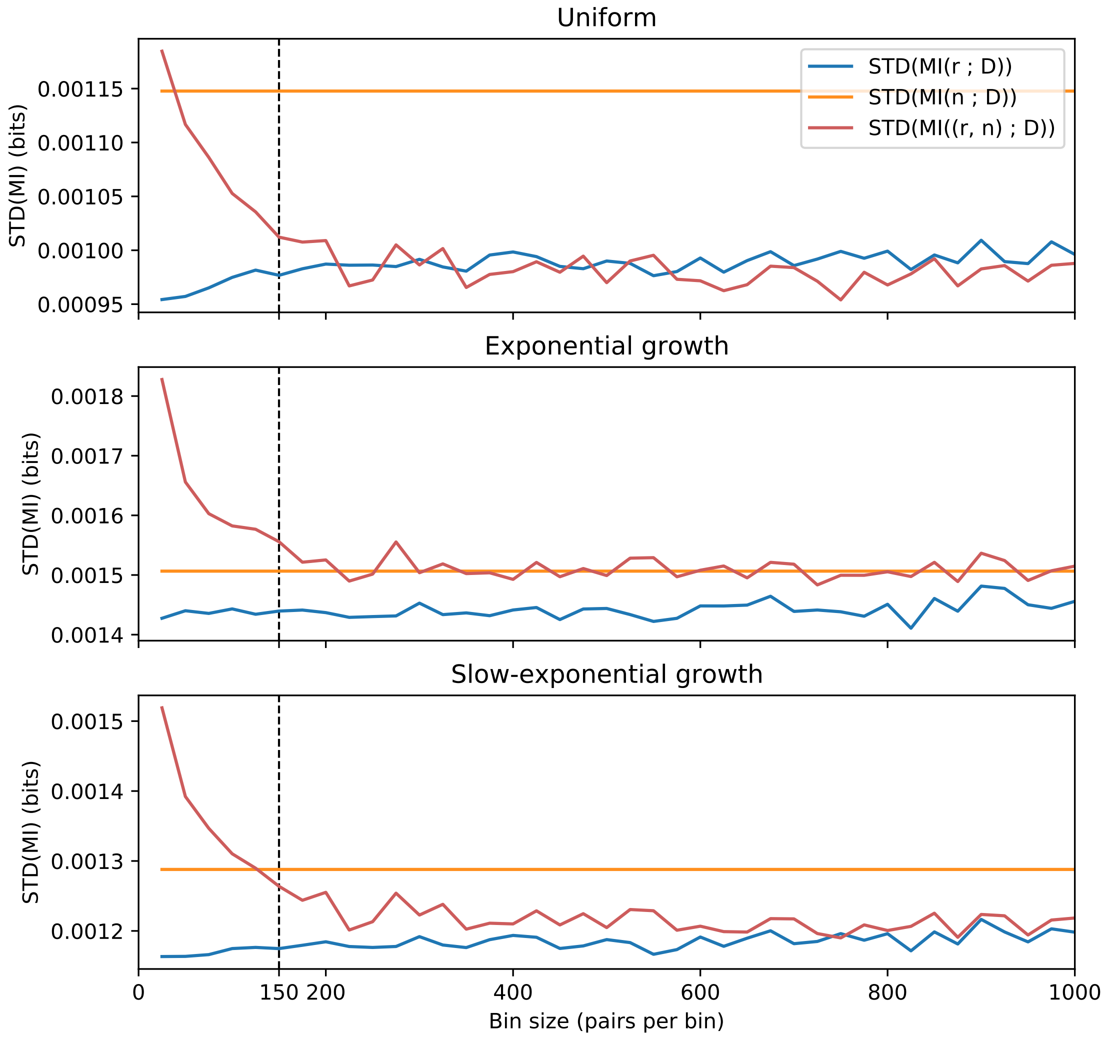

Figure S2: Standard deviations of MI of different feature sets as a function of bin size (pairs per bin), averaged over 80 independent simulations of exact segments from each of the distribution shapes.

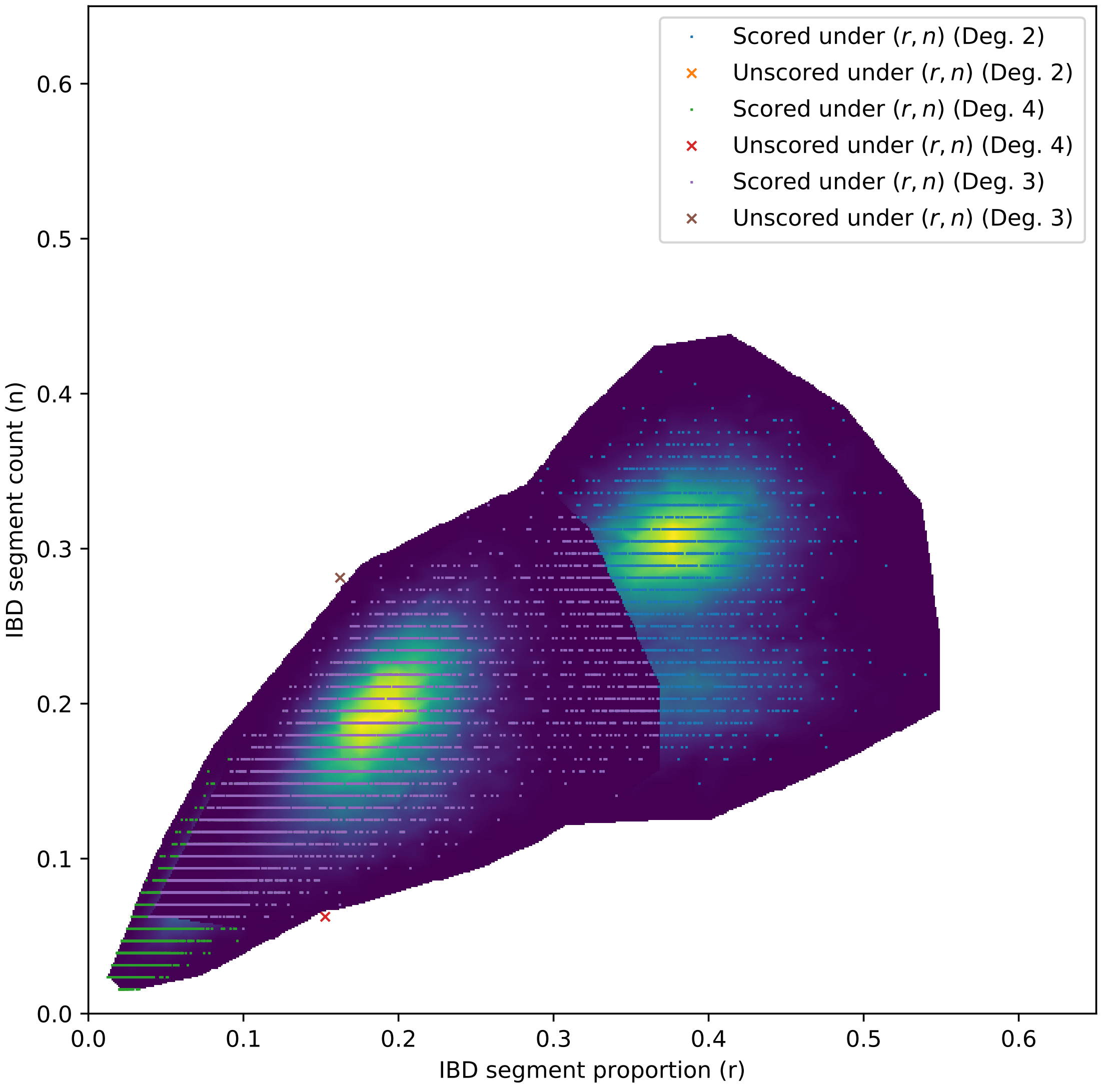

Figure S3: Heat maps depicting posteriors $\hat{p}(D|f,\vec{T})$ for inferred IBD segments for several values of D. Generated using the `griddata` two-dimensional interpolation on $\hat{p}(f|D)$ calculated from 210000 exact training data pairs. Overlaid are corresponding 21000 testing data points colored by their classification. Here, the IBD segment number n has been normalized to unity by dividing by the maximum segment count across all training data pairs. Probabilities and points from higher degrees are plotted on top of those from lower degrees.

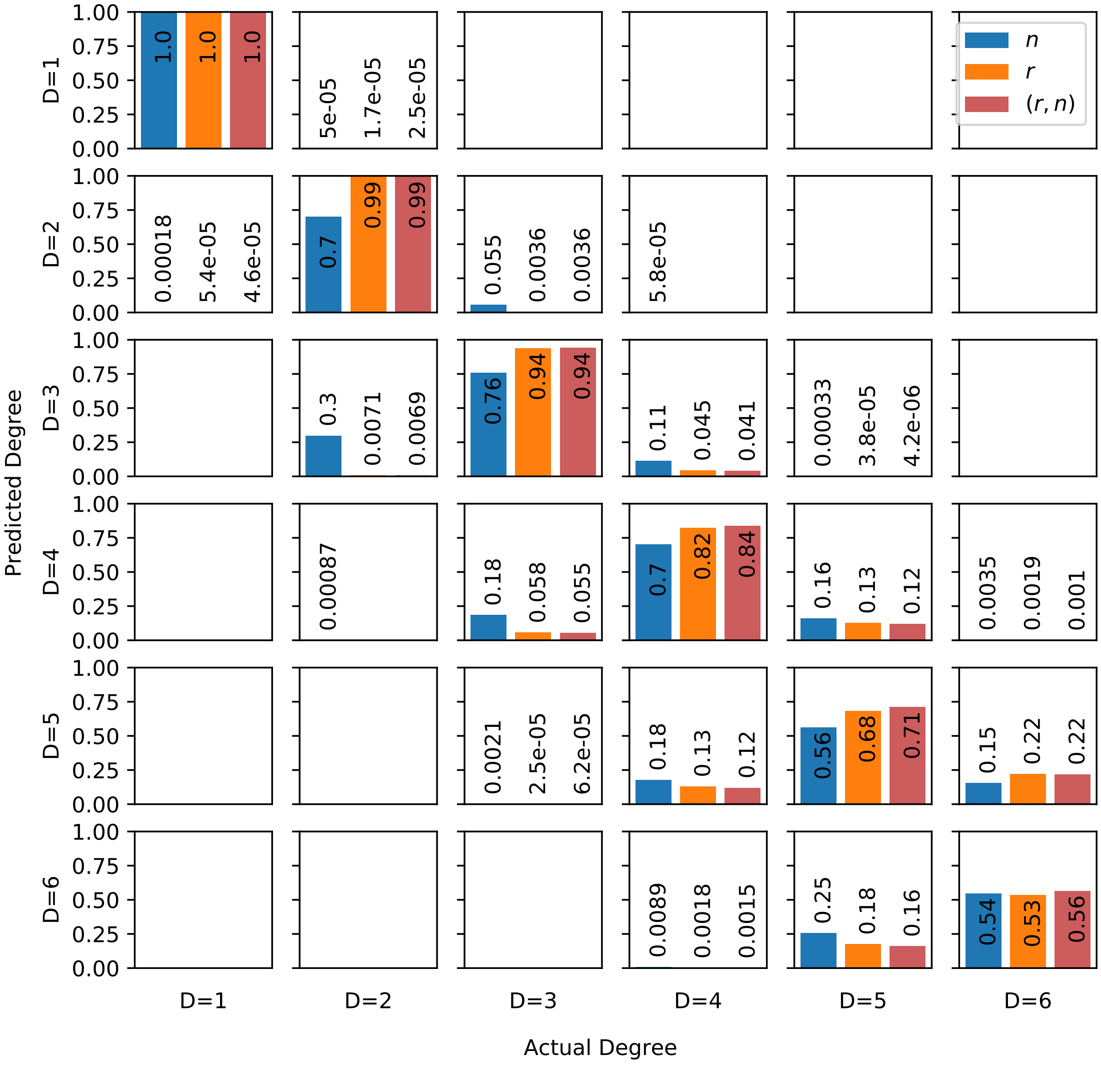

Figure S4: Confusion matrix (with respect to degree of relatedness) of Bayes classifiers trained on exact segments with features n, r and (r, n) from the uniform distribution. Most misclassifications occur in diagonal-adjacent cells (off-by-one-degree misclassifications).

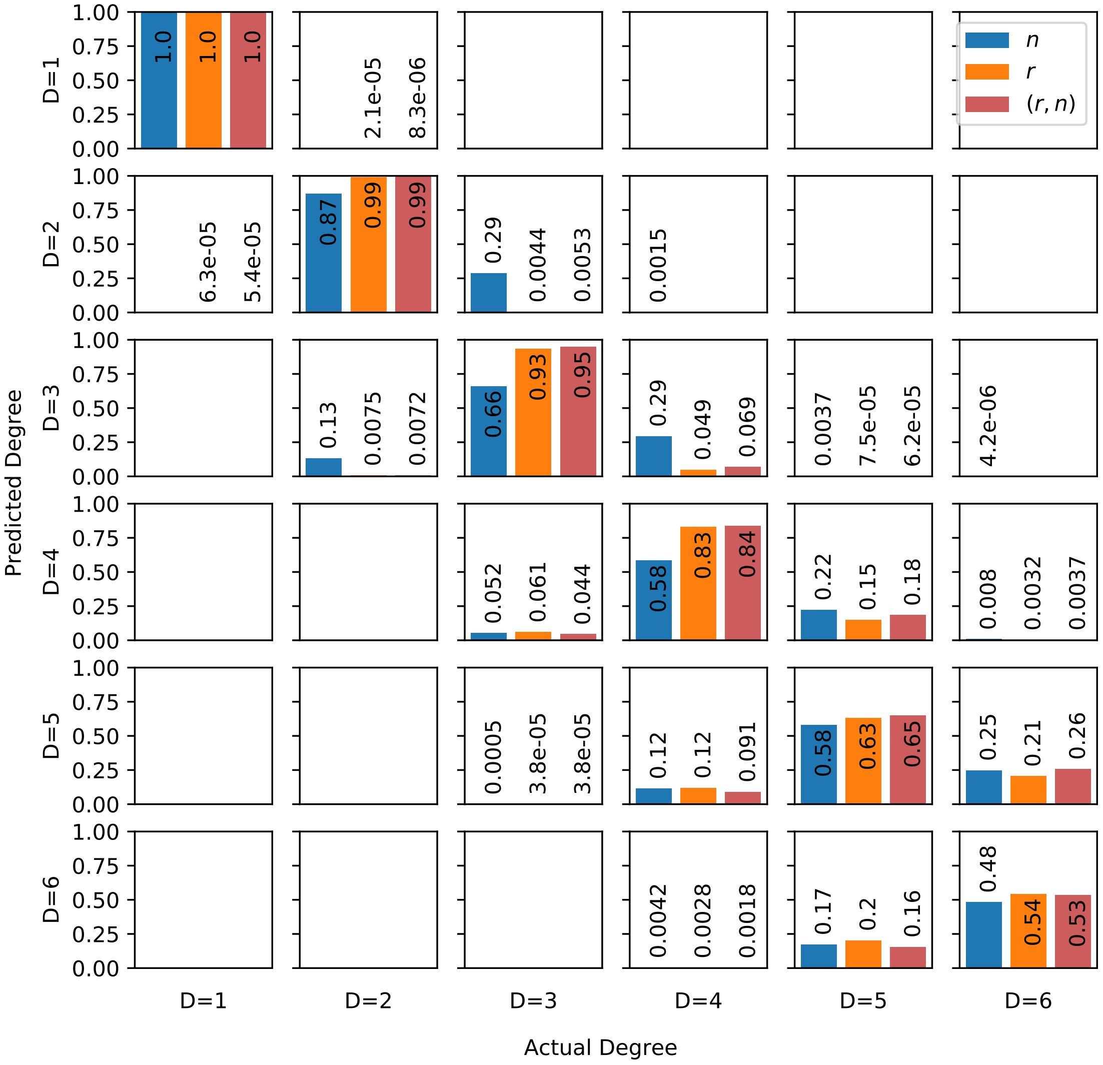

Figure S5: Confusion matrix (with respect to degree of relatedness) of Bayes classifiers trained on inferred segments with features n, r and (r, n) from the uniform distribution. Most misclassifications occur in diagonal-adjacent cells (off-by-one-degree misclassifications).

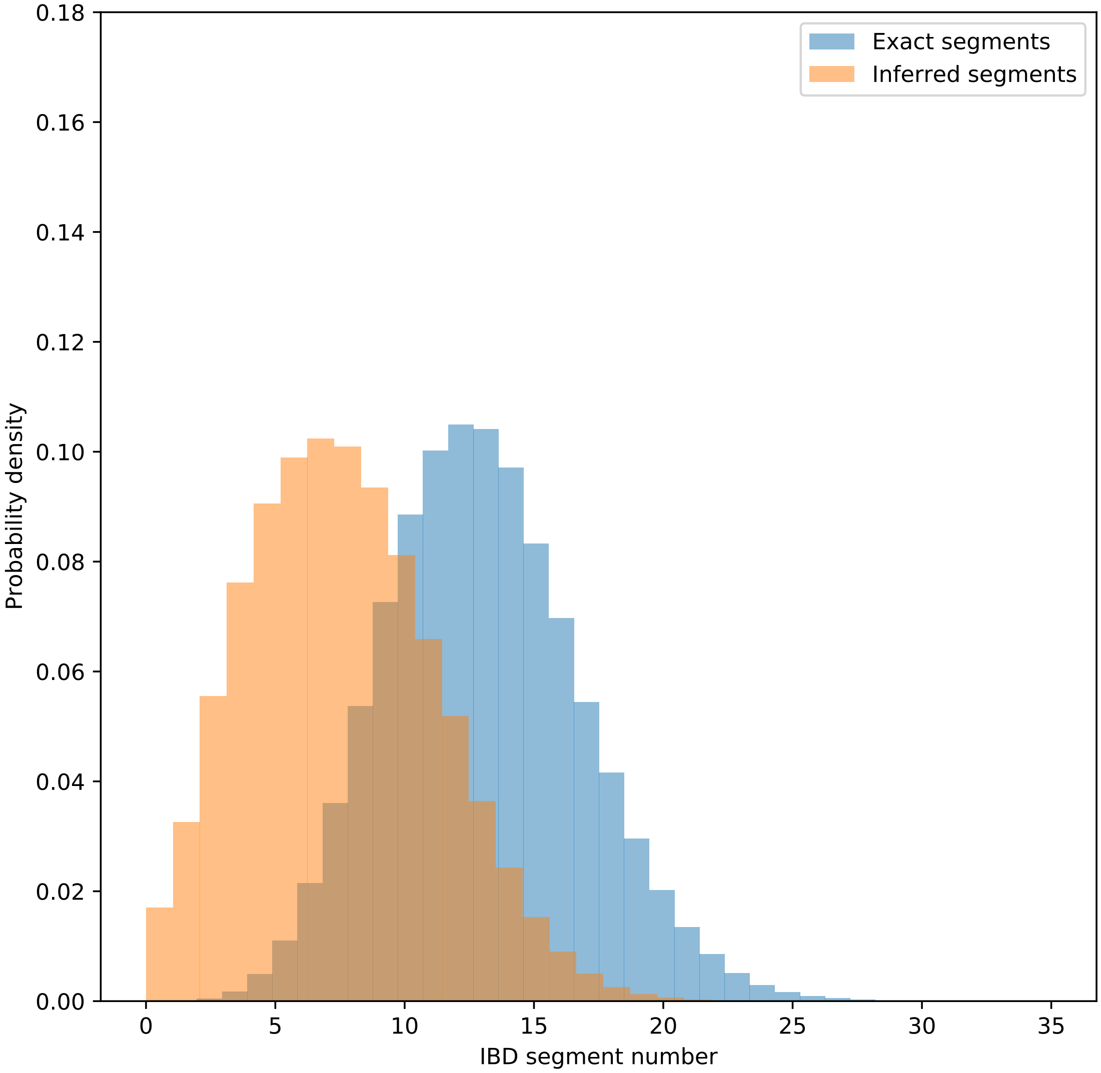

Figure S6: Distributions of exact and inferred segment numbers in fifth degree pairs.

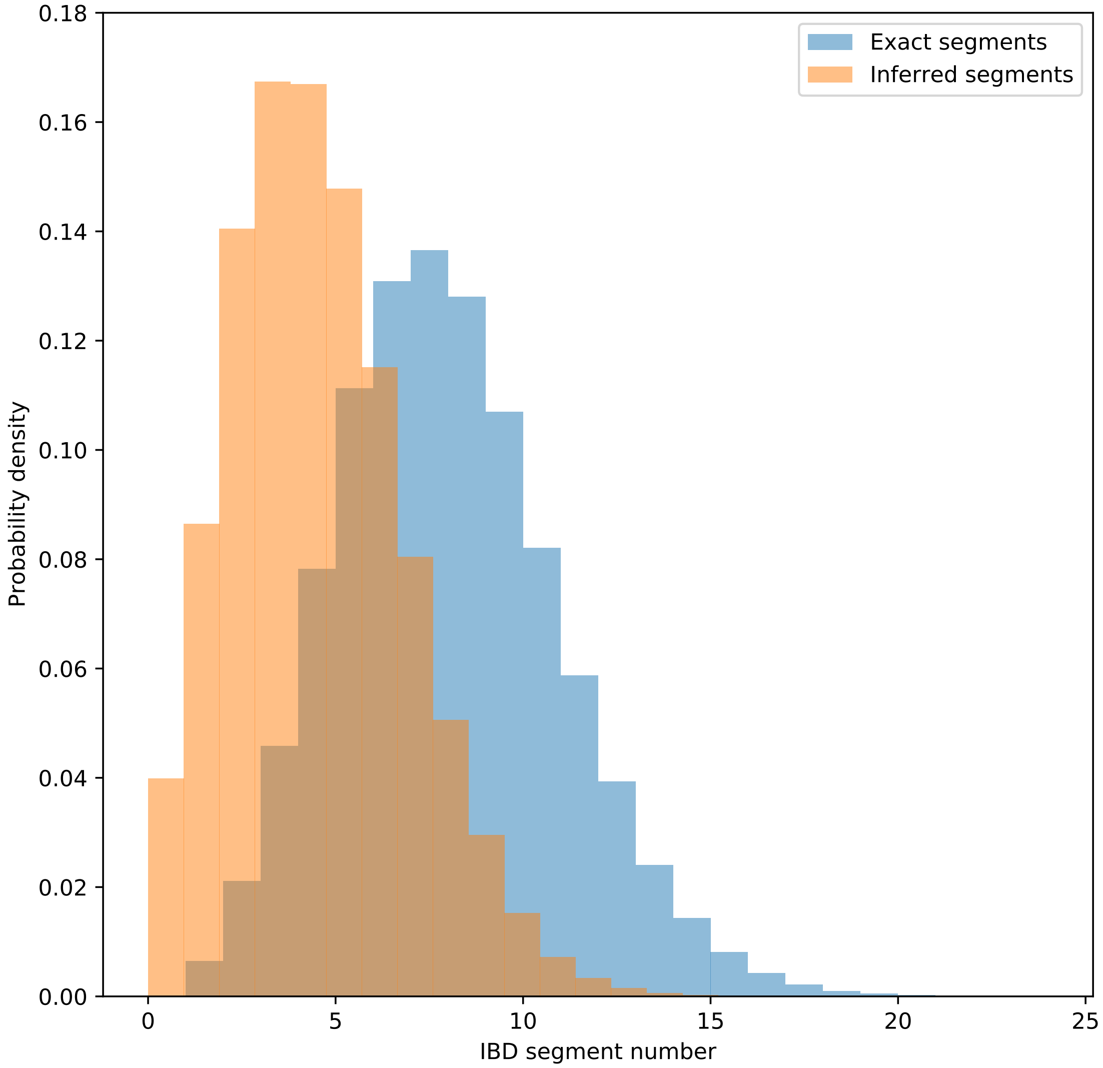

Figure S7: Distributions of exact and inferred segment numbers in sixth degree pairs.

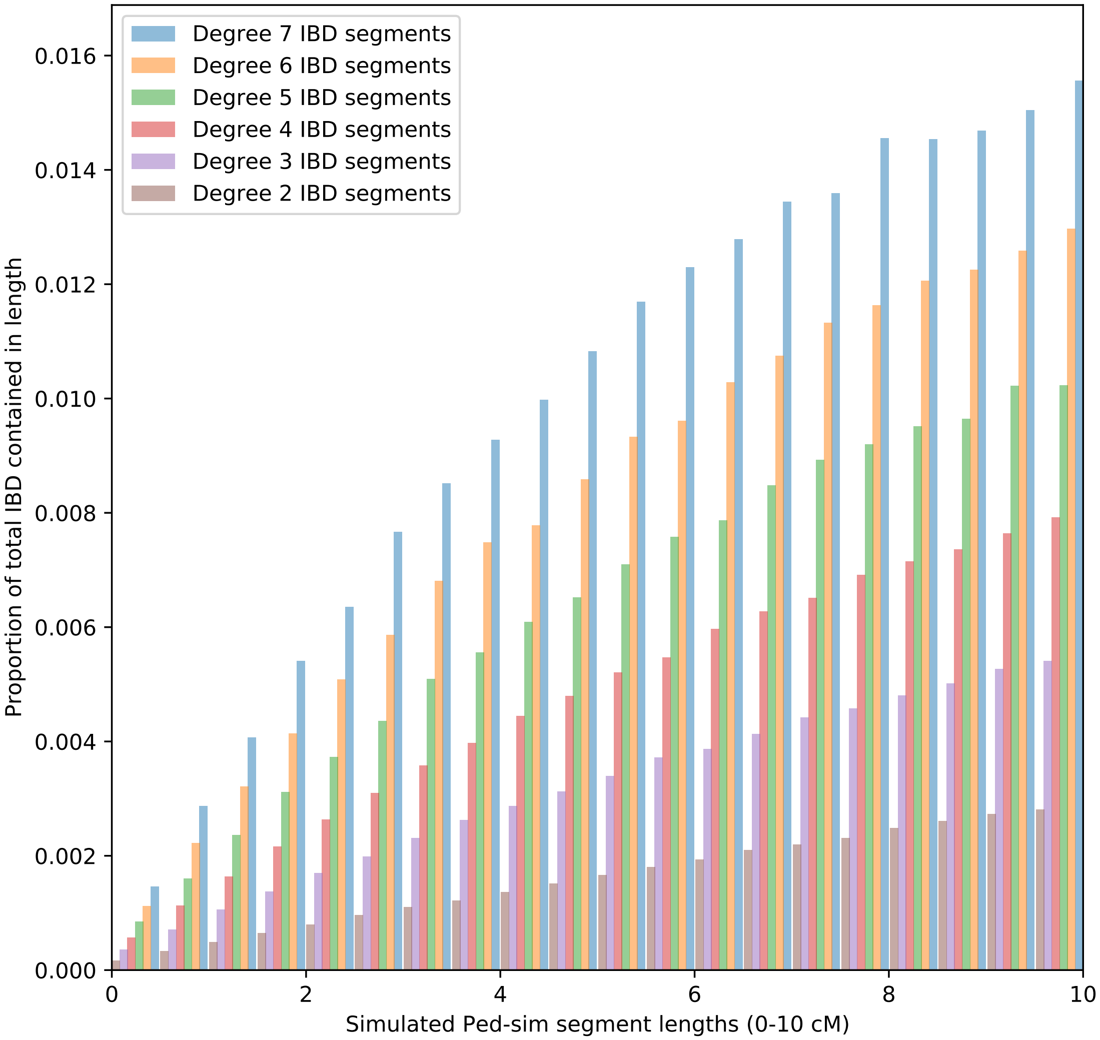

Figure S8: Average proportions of pairwise total IBD length contained in exact segments of lengths 0-10 cM for relatives of the indicated degrees. Proportions calculated over 33,000 pairs from each degree.
